# Calibration of Radar RCS Measurement Errors by Observing the Luneburg Lens Onboard the LEO Satellite

**DOI:** 10.3390/s22145421

**Published:** 2022-07-20

**Authors:** Jie Yang, Ning Li, Pengbin Ma, Bin Liu

**Affiliations:** 1 State Key Laboratory of Astronautic Dynamics, Xi’an Satellite Control Center, Xi’an 710043, China; nudtyang@163.com (J.Y.); map_bin@163.com (P.M.); liubin_astronomy@163.com (B.L.); 2 School of Electrical Engineering, Xi’an University of Technology, Xi’an 710048, China

**Keywords:** calibration, RCS measurement, radar RCS measurement errors, Luneburg Lens, LEO satellites

## Abstract

Accurate radar RCS measurements are critical to the feature recognition of spatial targets. A calibration method for radar RCS measurement errors is proposed for the first time in the context of special target tracking by observing the Luneburg Lens onboard the LEO satellite. The Luneburg Lens has favorable RCS scattering properties for the radar microwave. Thus, the laboratory RCS measurements of the Luneburg Lens, with some fixed incident frequency and with different incident orientations for the radar microwave, will be implemented in order to build a database. The incident orientation for the radar microwave in the satellite body frame will be calculated by taking advantage of the precise orbit parameters, with errors only at the magnitude of several centimeters and within the actual satellite attitude parameters. According to the incident orientation, the referenced RCS measurements can be effectively obtained by the bilinear interpolation in the database. The errors of actual RCS measurements can thus be calibrated by comparing the referenced and the actual RCS measurements. In the RCS measurement experiment, which lasts less than 400 s, the actual RCS measurement errors of the Luneburg Lens are nearly less than 0 dBsm, which indicates that the RCS measurement errors of the spatial targets can be effectively calculated by the proposed calibration method. After the elaborated calibration, the RCS measurements of the spatial targets can be accurately obtained by radar tracking.

## 1. Introduction

The radar cross section (RCS) is a physical measurement of the echo intensity generated by the target under the irradiation of a radar microwave. It is the imaging area of the target that is expressed by the projective area of an isotropic uniform-equivalent reflector, which has the same echo power as the target in the unit solid angle of the receiving direction [1]. The size of the target RCS depends on the following factors: the frequency of the incident electromagnetic wave (the wavelength of the electromagnetic wave which is the most important influencing factor), the incident orientation of the electromagnetic wave, the polarization mode of the incident electromagnetic wave, the target geometry, and the coating materials on the target surface [2,3]. For the stealth targets, the RCS is reduced mainly by optimizing the shape design. For the targets which are covered by the coating materials, the electromagnetic characteristics of the surface materials and the relationship between the direction of the radar microwave incident and the target position will reduce its RCS as a whole, and change its frequency and angle characteristics. Thus, RCS is a significant metric for these target features and its size can be utilized to distinguish different spatial targets.

Generally, the radar RCS measurement and its statistical characteristics are widely used in the feature recognition of spatial targets. The specific steps are described as follows: firstly, according to the known types of spatial targets, the target features are extracted from the RCS measurements; secondly, based on the corresponding relationship between the targets and the RCS characteristics, certain recognition criteria can be established; finally, the unknown targets are identified through the identification criteria. The RCS feature extraction method mainly focuses on the time domain and the transform domain. The extraction method in the time domain utilizes the periodic characteristics of the RCS measurement sequences [4]. The extraction method [3] in the transform domain includes the Fourier transform [5,6], wavelet transform [7,8], and the Merlin transform of the RCS measurement sequences [9]. Many methods have been attempted to establish the recognition criteria, such as the Bayesian method [10,11,12], the evidential reasoning method [13,14,15,16], the fuzzy classification method [17,18,19], and the neural network method [20,21,22,23,24]. The prior probability density function (PDF) distribution of targets, which are necessary to determine the minimum error rate or the minimum risk criterion, are requisites in the Bayesian method. If no prior information on targets can be obtained, it is usually assumed that the prior PDF distribution obeys the uniform distribution. In contrast, the evidential reasoning method does not employ the prior PDF distribution of targets. It can fuse the probability density distribution functions of different targets provided by different evidence and then determine the recognition criteria according to the new probability density distribution functions after fusion. The main idea of the fuzzy classification method is to transform the target features into fuzzy sets and member functions and then determine the target types through fuzzy relations and fuzzy reasoning. The neural network method has the abilities of self-adaptation, self-organization, and e-learning and it can deal with recognition problems in very complex environments or in some scenes with an unclear background. In this method, by constructing the sampling theory based on the training data, the unknown patterns are judged as the most recent memory.

For the target RCS to achieve this feature recognition, the multi-band RCS method is utilized wherein the RCS storage and measurements are compared in the frequency domain and the reversible discrete Fourier transform of RCS sequences is implemented in the time domain [25]. The target classification in the frequency domain is then realized by the nearest-neighbor decision rules. The automatic target recognition is performed by maximizing the correlation between observed and predicted values in the time domain. The complex targets are recognized by the wavelet transform of the RCS sequences measured by radar [26]. The orthogonal transformation of the RCS sequences which can reduce the computational complexity is implemented and then the aerial targets are successfully recognized [27]. The particle filter which employs the range and the RCS measurements in the MIMO radar network is utilized to achieve high-precision maneuvering target tracking [28]. After the discrete wavelet transform on the RCS sequences, five statistics, which can reflect the characters of the radar’s targets, are extracted and the set-valued model is proposed to describe the relationship between the feature vectors and the radar’s targets [29,30]. Through the simulation tests, it is found that higher target recognition accuracy can be obtained by this method than the fuzzy classification method and the evidential reasoning method.

The motion of the target and the radar cross-section are key parameters to be considered when designing a radar sensor for a given application. A supervised machine learning model (SVM) is trained using the recorded data to classify targets into four categories based on their radar cross-sections. The proposed non-contact radar combined with the SVM algorithm can be used to detect and classify targets in real time without the need for a signal processing toolbox [31]. A coherent integral detection algorithm based on dynamic programming (DP) and fractional Fourier transform (FrFT) is proposed. By combining the advantages of DP and FrFT, the proposed DP–FrFT method can rapidly search for target trajectories with simultaneous parameter estimation and motion compensation, thus achieving high integration gain with relatively low time consumption. The high efficiency of the method is verified by extensive simulations and adequate field experiments [32,33]. A new automatic target recognition (ATR) system and a complete ATR chain based on multidimensional features and a multilayer classifier system based on L-band holographic gaze radar are proposed [34]. However, the above-mentioned articles do not provide a detailed analysis and calibration of the radar RCS measurement errors.

Radar cross-section (RCS), as the above radar target feature recognition method, has become a significant characteristic quantity, which can be well applied to spatial target recognition. In the recognition process of spatial targets, it is necessary and vital to calibrate the RCS measurement errors. For spatial targets, the radar RCS measurement errors can be calibrated by observing the Luneburg Lens onboard the LEO satellites for the following two considerations. On the one hand, the Luneburg Lens has good scattering characteristics for any-direction incident radar microwave. On the other hand, the precise LEO orbit parameters with errors at the magnitude of several centimeters can be effectively utilized to calculate the inclination direction from the radar to the target [35].

The Luneburg lens has been widely used as the standard calibration source of RCS measurement errors in many ground and aerial calibration scenarios. However, the calibration of spatial targets has been rarely reported by the related literature. Essentially, the Luneburg lens is a synthetic multi-beam, large-capacity and wireless communication antenna [1]. As a wide-angle omni-directional antenna, it can transmit all kinds of large-angle incident electromagnetic waves back in parallel. Compared with other triangular reflectors, larger RCS and larger coverage angles of secondary radiation direction can be generated by the Luneburg lens, which indicates its obvious feature recognition abilities [1,36]. Thus, the scattering cross-section of the Luneburg lens carried on the low scattering trestle can be tested and calculated in a large-scale target characteristic laboratory. The database of referenced RCS can then be formed through the different incident directions of the radar microwave at multiple incident frequencies, usually including the P-band, L-band, S-band, C-band, X-band, and Ku-band frequencies. Meanwhile, the actual RCS of the Luneburg Lens onboard the LEO satellite is always yielded during the tracking by the radar at some fixed incident frequency. Finally, the radar RCS measurement errors can be calibrated by calculating the difference between the actual RCS measurements and the referenced RCS measurements in the database.

Besides, the line-of-sight direction from the radar to the spatial target, which is vital to the calibration procedure above, has to be calculated precisely based on the orbit parameters of the LEO satellite and the location parameters of the radar site. In addition to the line-of-sight direction, the satellite attitude parameters which describe the relationship between the satellite body frame and the orbital frame are also needed to calculate the projection of the line-of-sight direction on the Luneburg Lens. By means of the line-of-sight parameter and the attitude parameters, the referenced RCS can be calculated at different incident orientations, respectively.

The precise orbit parameters of LEO satellites are always calculated by the spaceborne highly dynamic GNSS receivers [37,38,39,40]. In 1992, the spaceborne GNSS receivers onboard the Topex/Poseidon radar altimetry satellites, which are jointly developed by the United States and France, were utilized to generate the GPS pseudo-range and carrier-phase observations. Based on these observations, the orbit determination with errors at the magnitude of several centimeters was achieved for the first time. Since then, a series of LEO satellites that are used in different scientific exploration missions have been equipped with GNSS receivers for precise orbit determination with the same orbit accuracy [41,42,43,44,45,46]. Therefore, the precise orbit parameters with errors of less than several centimeters are competent to calculate the line-of-sight parameter from the ground radar to the spaceborne Luneburg Lens. After transforming the line-of-sight direction from the orbital frame to the satellite body frame by the attitude parameters, the incident angles of the radar microwave can be obtained to calculate the referenced RCS in the RCS database. Thus, the actual RCS measurements can be compared with the referenced RCS measurements of the ground radar. By this means, the calibration of radar RCS measurement errors can be effectively fulfilled.

This paper is organized as follows: Section 2 describes the laboratorial RCS measurement principle of the spaceborne Luneburg Lens. The calibration method of the RCS measurement errors is illustrated in Section 3. The referenced RCS measurement results of the Luneburg lens in the laboratory tests are reported in Section 4. Section 5 gives the calibration results of actual RCS measurement errors. Section 6 summarizes the conclusion.

## 2. Laboratory RCS Measurement of Spaceborne Luneburg Lens

### 2.1. RCS Measurement Principle

The radar equation is the basis of the RCS measurements, which is usually expressed as follows [1]:

(1) $$\sigma =\frac{{\left(4\pi \right)}^{3}R^{4}P_{r}}{\lambda^{2}LG_{t}G_{r}P_{t}}$$

where $P_{r}$ denotes the echo power received by the radar, $P_{t}$ denotes the echo power transmitted by the radar, $G_{r}$ denotes the gain of the radar receiving antenna, $G_{t}$ denotes the gain of the radar transmitting antenna, λ denotes the radar wavelength, σ denotes the RCS of the target, R denotes the distance between the radar and the target, and L denotes the system loss. In the static test field wherein the parameters of the measurement system such as the frequency, polarization, antenna gain, transmission power, and test distance hold the same, the RCS of different targets in the same test conditions can be distinguished only by the parameter of $P_{r}$. Thus, once the received echo power of some referenced target with known RCS of $\sigma_{\mathrm{Ref}}$ is obtained, the RCS of unknown targets can be readily calculated by the following ratio:

(2) $$\sigma_{\mathrm{Target}}=\frac{P_{r,\mathrm{Target}}}{P_{r,\mathrm{Ref}}}\sigma_{\mathrm{Ref}}$$

where $P_{r,\mathrm{Ref}}$ denotes the received echo output of the referenced target and $P_{r,\mathrm{Target}}$ denotes the received echo output of unknown targets.

Therefore, the measurement value of RCS can be obtained by measuring the reference body to obtain its echo response and then placing the target body to obtain its echo response.

### 2.2. RCS Indoor Test Procedure

**Figure 1:**
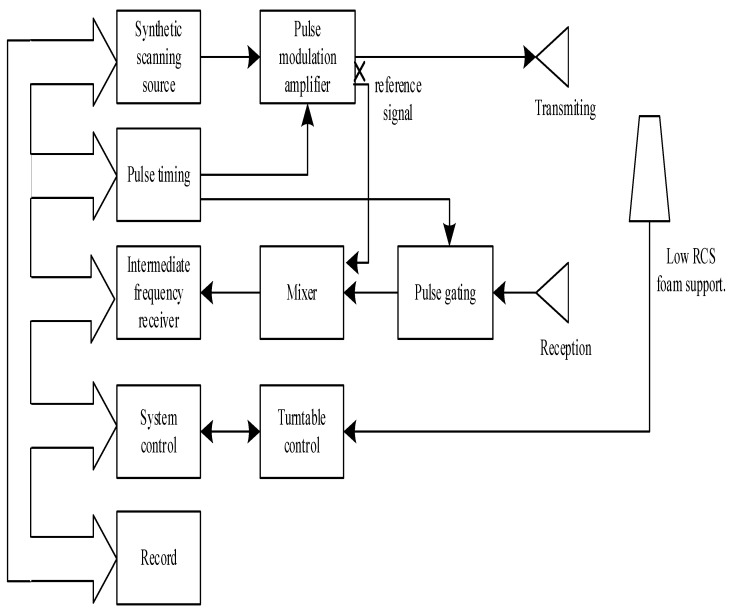
The block diagram of the RCS measurement system.

The RCS indoor measurement system consists of the following: a receiving and transmitting antenna subsystem, a transmitting subsystem, a receiving subsystem, a turntable control subsystem, and a data acquisition and processing subsystem. The simplified block diagram is shown in Figure 1.

In order to meet the requirements of circular polarization test for phase accuracy, the test mode of one transmitter and two receivers in broadband is realized through a multi-channel parallel test and polarization switch. In other words, as the H or V polarization is transmitted, both the H and V are received at the same time. Thus, this procedure not only improves the test efficiency, but also ensures the stability of the scattering center phase in different polarization tests and enhances the precision of synthesizing circular polarization from linear polarization.

The specific test procedure is briefly described, as follows:

Step 1: Calibrate the rhombic dihedral angle to obtain the echo responses of the horizontally polarized transmission and the horizontally polarized reception of the rhombic dihedral angle and its empty chamber.

Step 2: Measure the echo responses of the horizontally polarized transmission and the horizontally polarized reception of the Luneburg Lens with a 200 mm diameter, calibrate full polarization with the echo data of Step 1, synthesize the linear polarization into circular polarization, and obtain the echo responses of the horizontally polarized transmission and the horizontally polarized reception of the Luneburg Lens, and then compare them with the theoretical values. If the difference between them is less than 0.3 dB, go to Step 3.

Step 3: Test the echo responses of the horizontally polarized transmission and reception of the combination of the Luneburg Lens and the satellite, calibrate full polarization with the echo data of Step 1, and then synthesize the linear polarization into circular polarization to obtain the horizontally polarized transmission and reception echo responses of the combination.

## 3. Calibration Method of the RCS Measurement Errors

### 3.1. Frame and Parameter Definition

Earth-centered inertial frame (ECI): The origin is at the Earth’s barycenter. The $x_{i}$ axis points to the vernal equinox at some reference epoch. The $z_{i}$ axis is along the axis of the Earth’s rotation through the conventional terrestrial pole (CTP). The $y_{i}$ axis forms a right-handed orthogonal system.

Earth-centered Earth-fixed frame (ECEF): The Earth-fixed coordinate system is a fixed coordinate system with the center of the Earth as its origin. Its $x_{e}$ axis points to the intersection of the equatorial plane and Greenwich meridian, its $z_{e}$ axis points to the CTP, and its $y_{e}$ axis, $z_{e}$ axis, and $x_{e}$ axis form a right-handed orthogonal system.

Satellite body frame: The origin is at the center of the satellite mass. The $z_{b}$ axis points to the Luneburg Lens. The $y_{b}$ axis points to the normal direction which is perpendicular to the solar wing. The $x_{b}$ axis forms a right-handed orthogonal system with the $y_{b}$ and $z_{b}$ axes.

Orbital frame: The origin is at the satellite centroid. The opposite direction of the $y_{o}$ axis points to the normal line of the orbital plane, the $z_{o}$ axis points to the geocentric direction, and the $x_{o}$ axis obeys the right-handed orthogonal convention.

**Figure 2:**
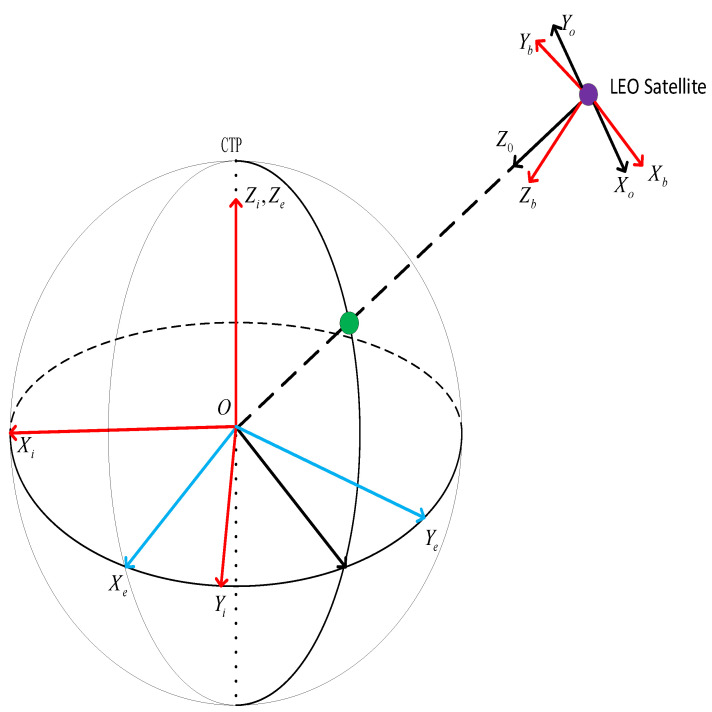
The graphical description of different frames.

The relationship between the above frames is graphically shown in Figure 2.

Satellite attitudes: The satellite attitudes represent the relationship between the orbit frame and the satellite body frame. The rotations from the orbital frame to the satellite frame are defined as the first rotation around the *z*-axis, followed by the rotation around the *x*-axis and, finally, the rotation around the *y*-axis. The corresponding attitudes are defined as yaw, roll, and pitch, respectively.

**Figure 3:**
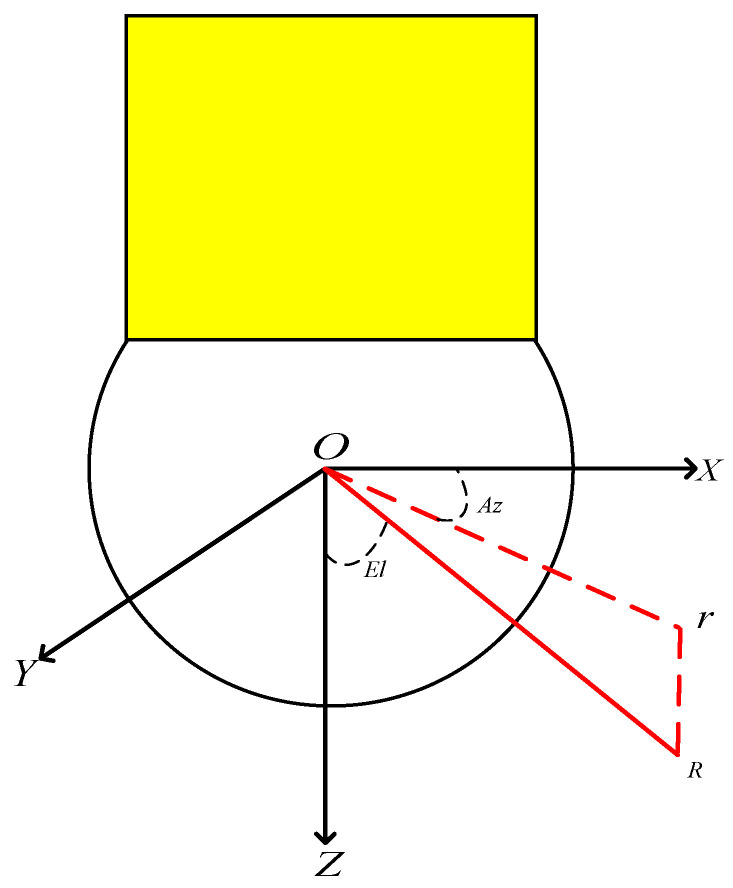
The radar microwave incident angles.

Radar microwave incident direction: The incident direction of the radar microwave is described by elevation and azimuth in the satellite body frame. The azimuth denotes the intersection angle between the projection of the line-of-sight direction on the *x*-*y* plane and the *x*-axis direction in the satellite body frame, which is zero as it coincides with the *x*-axis direction and positive as it rotates around the *z*-axis. The elevation denotes the intersection angle between the line-of-sight direction and the *z*-axis direction in the satellite body frame, which is zero as it coincides with the *z*-axis direction and π/2 as it coincides with the *x*-*y* plane (as shown in Figure 3).

### 3.2. Calculation of Satellite Centroid Position Vector

Given that the position and velocity vectors of a point of N on the satellite body is $P_{s}$ and $V_{s}$ in the ECEF frame, and the position vector of this point is $P_{sc}$ in the satellite body frame, calculate the position vector of the satellite centroid of $P_{o}$ in the ECEF frame.

Assuming the attitudes of the three-axis stable satellite are nearly constant and the influence of attitude angular velocity can be ignored, the velocity vector of this point in the ECI frame is $V_{si}$, which is expressed as

(3) $$V_{si}=V_{s}+\omega \times P_{s}$$

where $\omega ={\left[\begin{array}{ccc} 0 & 0 & \omega_{e} \end{array}\right]}^{T}$, and $\omega_{e}$ denotes the angular rate of the Earth’s rotation.

The direction vectors of three axes in the orbital frame are expressed as follows:

(4) $$\left\{\begin{array}{l} V_{orb}^{z}=-P_{s}/\sqrt{P_{s}\cdot P_{s}}\\ V_{orb}^{y}=\left(V_{orb}^{z}\times V_{si}\right)/\sqrt{\left(V_{orb}^{z}\times V_{si}\right)\cdot \left(V_{orb}^{z}\times V_{si}\right)}\\ V_{orb}^{x}=V_{orb}^{y}\times V_{orb}^{z} \end{array}\right.$$

According to Equation (4), the transformation matrix from the orbital frame to the ECEF frame can be expressed as

(5) $$C_{orb}^{ecef}=\left[\begin{array}{ccc} V_{orb}^{x} & V_{orb}^{y} & V_{orb}^{z} \end{array}\right]$$

Define the roll as φ, the pitch as θ, and the yaw as ψ, then the transformation matrix from the satellite body frame to the orbital frame is expressed as

(6) $$C_{sat}^{orb}=\left(\begin{array}{ccc} c\psi c\theta -s\theta s\psi s\varphi & -s\psi c\varphi & c\psi s\theta +s\psi s\varphi c\theta \\ s\psi c\theta +c\psi s\varphi s\theta & c\psi c\varphi & s\psi s\theta -c\psi s\varphi c\theta \\ -c\varphi s\theta & s\varphi & c\varphi c\theta \end{array}\right)$$

where c denotes the cosine operation and s denotes the sine operation.

Thus, the position vector of $P_{o}$ in the ECEF frame is calculated as

(7) $$P_{o}=P_{s}-C_{orb}^{ecef}C_{sat}^{orb}P_{sc}$$

### 3.3. Calculation of the Radar Microwave Incident Direction

Given the position vectors of the radar and the satellite in the ECEF frame are $P_{z}$ and $P_{s}$, respectively, the line-of-sight direction from the radar to the satellite in the ECEF frame is expressed as

(8) $$V_{los}^{ecef}=\left(P_{s}-P_{z}\right)/\sqrt{\left(P_{s}-P_{z}\right)\cdot \left(P_{s}-P_{z}\right)}$$

Thus, the line-of-sight direction in the satellite body frame can be readily obtained as

(9) $$V_{los}^{sat}=C_{orb}^{sat}C_{ecef}^{orb}V_{los}^{ecef}={\left(C_{sat}^{orb}\right)}^{T}{\left(C_{orb}^{ecef}\right)}^{T}V_{los}^{ecef}$$

According to the line-of-sight direction in the satellite body frame, the incident elevation of the radar microwave is expressed as

(10) $$El=\cos^{-1}\left(V_{los}^{sat}\cdot V_{z}\right)$$

where $V_{z}={\left[\begin{array}{ccc} 0 & 0 & 1 \end{array}\right]}^{T}$.

Define the temporary vector of $V_{m}$ in the satellite body frame as follows:

(11) $$V_{m}=\left(V_{los}^{sat}\times V_{z}\right)/\sqrt{\left(V_{los}^{sat}\times V_{z}\right)\cdot \left(V_{los}^{sat}\times V_{z}\right)}$$

Thus, calculate the temporary angle of H as

(12) $$H=\cos^{-1}\left(V_{m}\cdot V_{x}\right)$$

where $V_{x}={\left[\begin{array}{ccc} 1 & 0 & 0 \end{array}\right]}^{T}$.

Then, the incident azimuth of the radar microwave is expressed as follows:

(13) $$Az=\left\{\begin{array}{ll} H+\pi /2, & if\;V_{m}\left(2\right)\geq 0\\ \pi /2-H, & if\;V_{m}\left(2\right)<0\;and\;H\leq \pi /2\\ 5\pi /2-H, & if\;V_{m}\left(2\right)<0\;and\;H>\pi /2 \end{array}\right.$$

### 3.4. Calculation of Referenced RCS

According to the incident elevation and azimuth of the radar microwave at some fixed incident frequency, the referenced RCS can be searched in the RCS database which has been obtained by the laboratory tests. For one set of azimuth and elevation at some observation epoch, namely Az and El, the referenced RCS can be readily calculated by the following bilinear interpolation method:

(14) $$\begin{array}{cc} RCS & =\frac{{RCS}_{i,i}(Az-Az_{i+1})(El-El_{i+1})}{\left(Az_{i+1}-Az_{i})(El_{i+1}-El_{i}\right)}\\ & +\frac{{RCS}_{i+1,i}(Az-Az_{i})(El_{i+1}-El)}{\left(Az_{i+1}-Az_{i})(El_{i+1}-El_{i}\right)}\\ & +\frac{{RCS}_{i,i+1}(Az_{i+1}-Az)(El-El_{i})}{\left(Az_{i+1}-Az_{i})(El_{i+1}-El_{i}\right)}\\ & +\frac{{RCS}_{i+1,i+1}(Az-Az_{i})(El-El_{i})}{\left(Az_{i+1}-Az_{i})(El_{i+1}-El_{i}\right)}\\ & Az\in [Az_{i},Az_{i+1}],El\in [El_{i},El_{i+1}] \end{array}$$

where $RCS_{i,i}$ denotes the referenced RCS at the elevation of $El_{i}$ and the azimuth of $Az_{i}$. The explanation of $RCS_{i,i+1}$, $RCS_{i+1,i}$, and $RCS_{i+1,i+1}$ are analogous to $RCS_{i,i}$. According to Equation (14), the actual RCS measurement can be compared with the referenced RCS at some observation epoch.

## 4. Laboratory RCS Measurement Results of the Luneburg Lens

**Figure 4:**
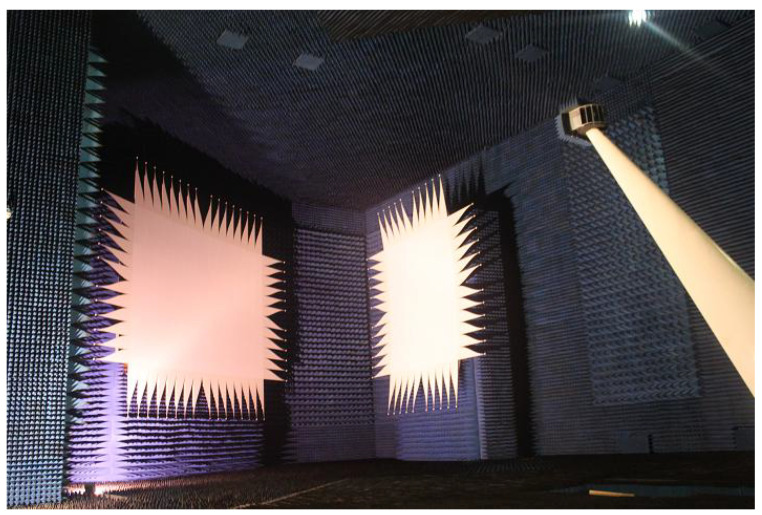
A large-scale target-characteristic laboratory.

The incident frequency of the radar microwave is set as 3.3 GHZ in the laboratory test, which is implemented in a large darkroom as shown in Figure 4. The horizontal polarization mode is both adopted by the transmitting and receiving antenna. The background RCS is less than −50 dBsm which can be deemed as no interference to the target RCS measurements.

**Figure 5:**
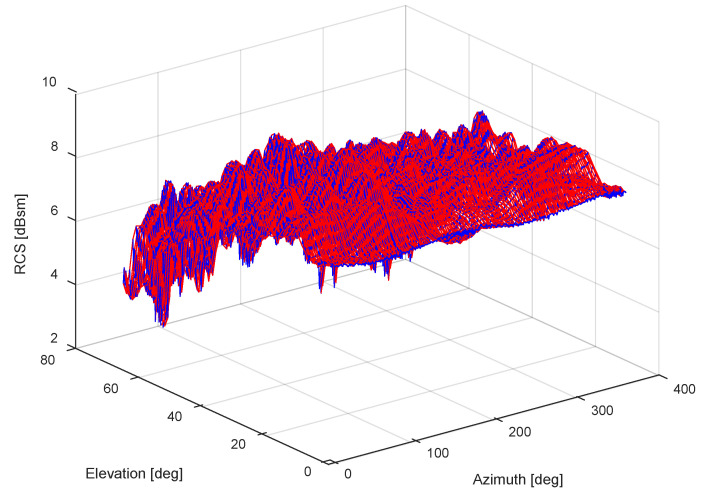
The database of laboratory RCS measurements.

The RCS measurement results of the Luneburg Lens at different incident angles (total counts to 360 × 66 = 23,760 points), namely the azimuths from 0° to 359° and the elevation from 0° to 65° both uniformly spaced by the angle of 1°, are shown in Figure 5. The RCS measurement errors at each set of azimuth and elevation is less than 0.4 dBsm which can be accepted for the more than 4 dBsm RCS measurement itself.

**Figure 6:**
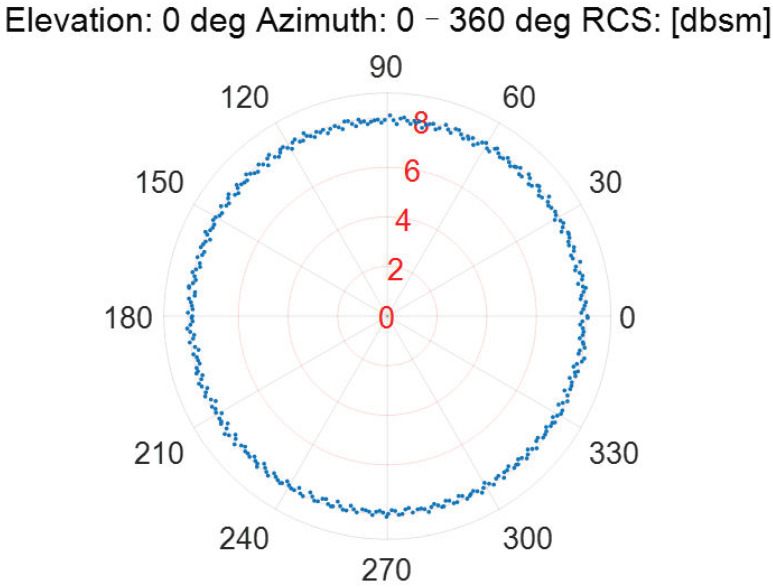
The RCS measurement results at the elevation of 0 deg.

**Figure 7:**
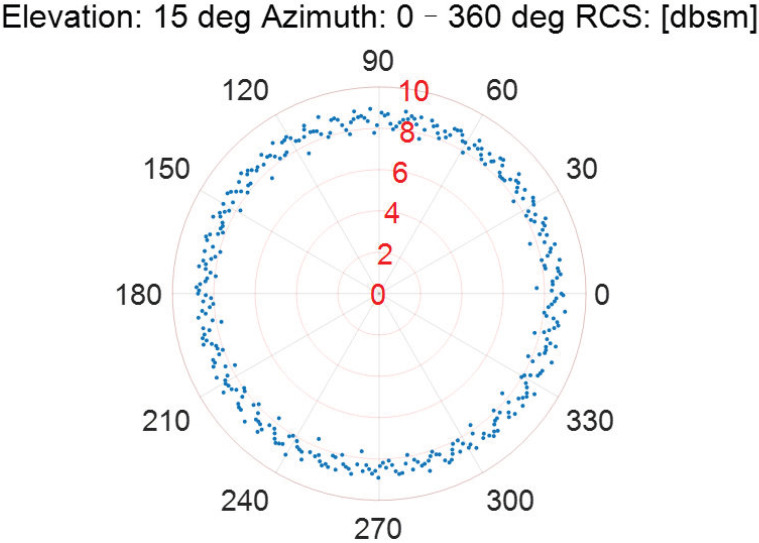
The RCS measurement results at the elevation of 15 deg.

**Figure 8:**
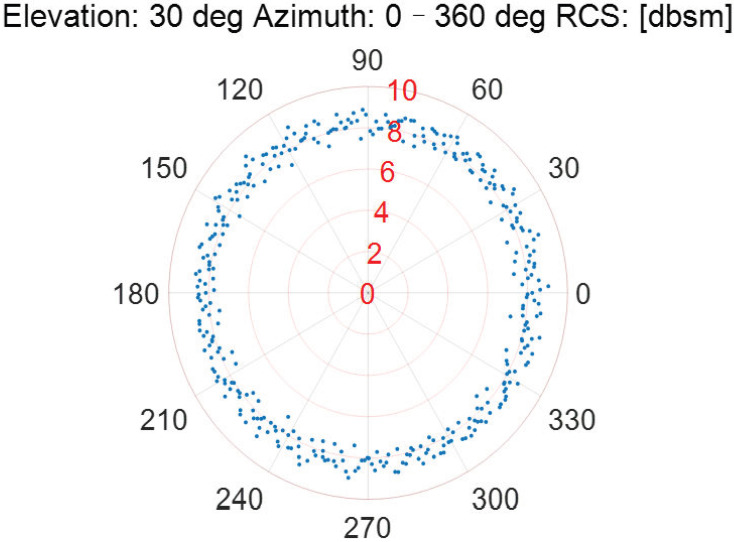
The RCS measurement results at the elevation of 30 deg.

**Figure 9:**
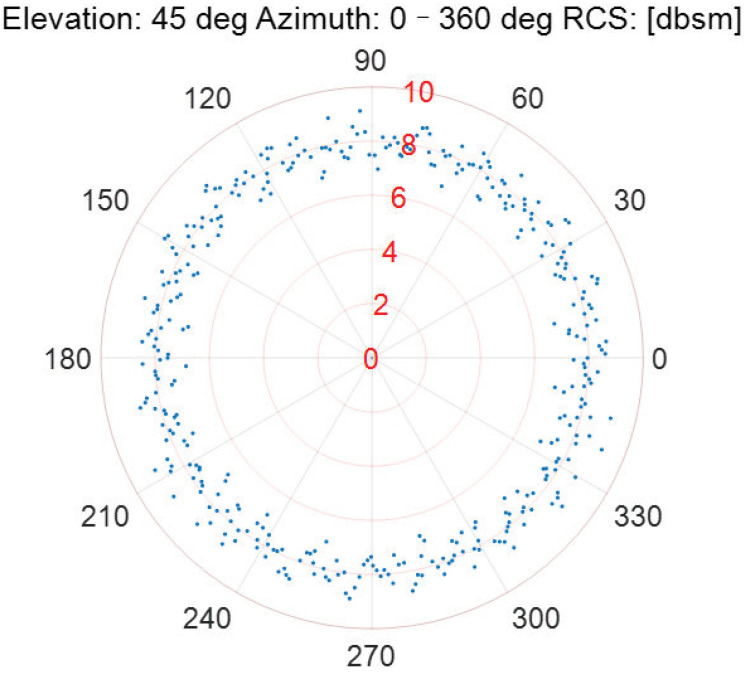
The RCS measurement results at the elevation of 45 deg.

**Figure 10:**
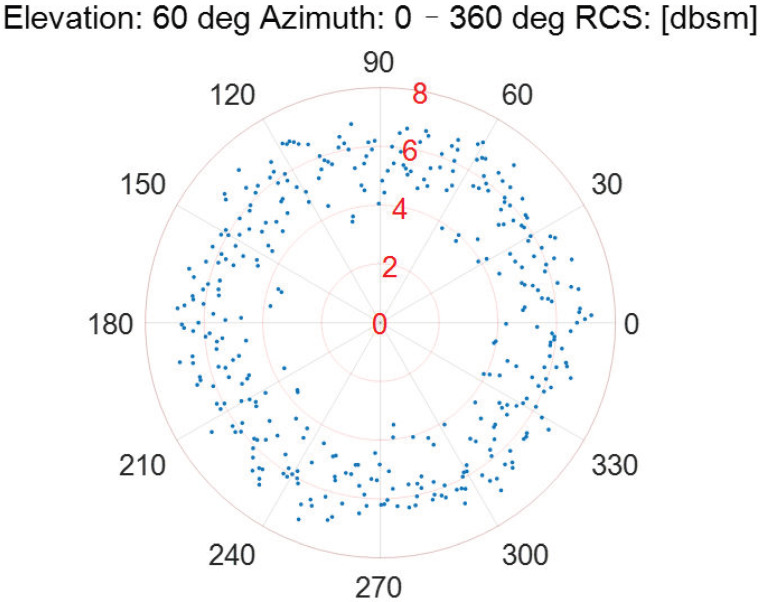
The RCS measurement results at the elevation of 60 deg.

As the incident elevation is fixed, the RCS measurement results of the Luneburg Lens at different incident azimuths are described in Figure 6, Figure 7, Figure 8, Figure 9 and Figure 10.

According to Figure 6, Figure 7, Figure 8, Figure 9 and Figure 10, the referenced RCS measurements of the Luneburg Lens are nearly at the same magnitude for the incident radar microwave, with some fixed elevation and arbitrary azimuth. However, the RCS measurement errors are closely related with the incident angles, including not only the elevation but also the azimuth. In fact, the RCS measurement errors at each set of azimuth and elevation are less than 0.4 dB in the laboratory tests. Thus, the Luneburg Lens has good scattering characteristics for the radar microwave, which can be well utilized as the calibration source.

## 5. Calibration Results of Actual RCS Measurement Errors

In tracking the Luneburg Lens onboard the LEO satellite by the ground radar, the same incident frequency as the laboratory RCS measurements is set for the RCS measurement experiment. The initiative tracking occasion is at UTC epoch 2021.12.11 09:16:56.75 s. The RCS measurement period is 0.05 s.

**Figure 11:**
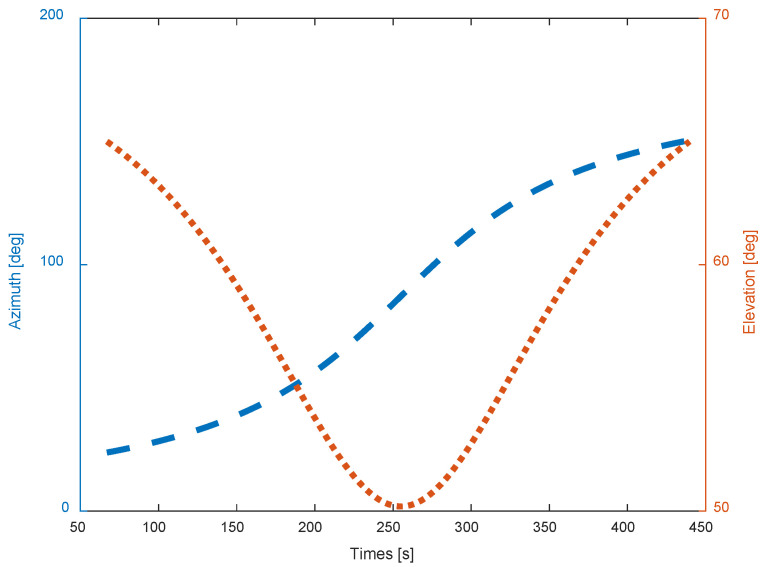
The incident orientation of the radar microwave in tracking the spaceborne Luneburg Lens by a ground radar.

During the tracking procedure which lasts about 372 s, the incident azimuth and elevation of the radar microwave are described in Figure 11. As is clearly illustrated, the elevation lies in the 50–65-degree scope and the azimuth in the 15–150-degree scope. The incident angles are obviously included in the predetermined database. Then, the accurate referenced RCS can be calculated by the bilinear interpolation method.

**Figure 12:**
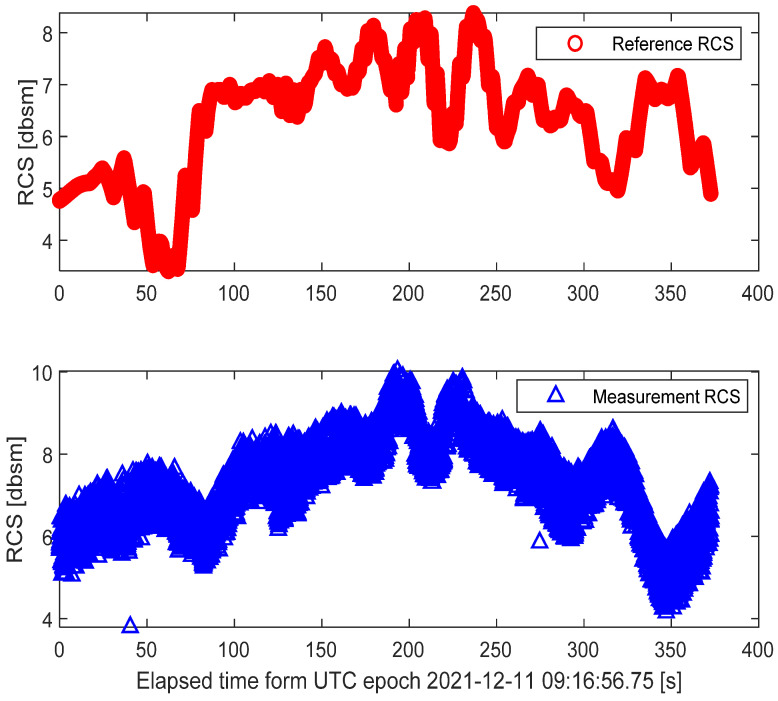
Comparison of the actual RCS measurement and the referenced RCS.

**Figure 13:**
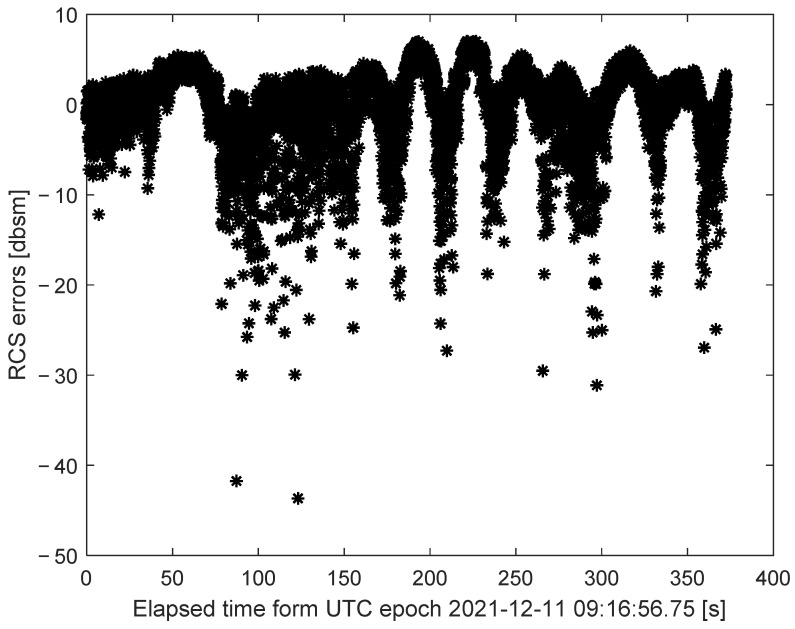
The radar RCS measurement errors denoted by * during the tracking procedure.

**Figure 14:**
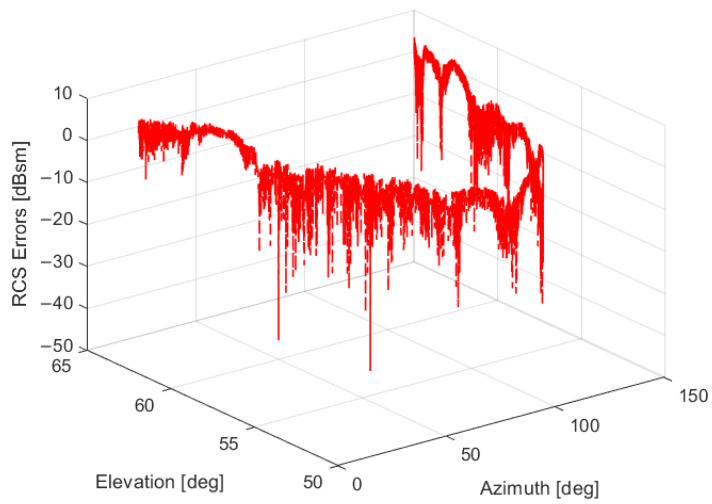
The radar RCS measurement errors at different incident angles.

The referenced RCS and measurement RCS for the whole tracking procedure are compared in Figure 12. According to the radar RCS calibration method in Section 3, by comparing the actual radar RCS measurements with the referenced RCS, the RCS measurement errors can be clearly depicted in Figure 13 and Figure 14.

As is illustrated in Figure 13 and Figure 14, the radar RCS measurement errors of the spaceborne Luneburg Lens are almost less than 0 dBsm in most tracking periods. These calibration errors of the RCS measurements can be well utilized to evaluate the RCS measurement performance of the radar. After correcting the RCS measurement errors of the specified ground radar, better RCS measurement properties can be guaranteed during its tracking of the other spatial targets.

## 6. Conclusions

In order to improve the radar RCS measurement accuracy and realize the feature recognition of spatial targets based on more accurate RCS measurements, this paper proposes a calibration method of radar RCS measurement errors based on observing the spaceborne Luneburg Lens. The main contribution of this paper is summarized as follows:

(1) The referenced RCS of the Luneburg Lens at different incident frequencies and the incident direction of the radar microwave are adequately measured in a point-by-point fashion in a large-scale target-characteristic laboratory in order to build a database. The database covers an incident angle spectrum with a 0–359-degree azimuth and a 0–65-degree elevation. Such an extensive database is enough to provide the referenced RCS to most LEO target tracking.
(2) The RCS calculation method of spatial targets is proposed mainly by calculating the line-of-sight direction from the radar to the Luneburg Lens. Some necessary frame transforms are essentially needed. First, the precise orbit parameters of the LEO satellite at the magnitude of several centimeters and the radar’s location parameters are combined to calculate the line-of-sight parameters in the ECEF frame, followed by the change to its satellite body frame counterpart with the satellite attitude parameters. Then, the incident elevation and azimuth of the radar microwave can be successfully calculated by the line-of-sight components in the satellite’s body frame. Thus, the referenced RCS measurements at different observation epochs are calculated by the bilinear interpolation in the predetermined database.
(3) A radar tracking the Luneburg Lens onboard a LEO satellite is schemed in the timespan of less than 400 s to obtain its actual RCS measurements. The RCS measurement errors at some observation epochs are readily evaluated by comparing the actual RCS measurements with the referenced RCS.

According to the well-designed tracking test, the RCS measurement errors in the tracking periods of a ground radar are almost less than 0 dBsm, which indicates that the calibration method of RCS measurement errors based on observing the Luneburg Lens onboard the LEO satellite can provide the accurate correction for the actual RCS measurements of ground radars at different incident frequencies. After correcting the RCS measurement errors, the radar can track the spatial targets to provide more accurate RCS measurements which are vital to the feature recognition in the sequel.

## Data Availability

Some or all data, models, or code that support the findings of this study are available from the corresponding author upon reasonable request. Some or all data, models, or code generated or used during the study are proprietary or confidential in nature and may only be provided with restrictions.
